# Ophthalmic screening in phakomatoses

**DOI:** 10.3389/fcell.2026.1785180

**Published:** 2026-07-03

**Authors:** Bijal Kikani, Sarah Stanley, Aparna Ramasubramanian

**Affiliations:** 1 Medical College of Wisconsin, Milwaukee, WI, United States; 2 SUNY Upstate Medical University, Norton College of Medicine, Syracuse, NY, United States

**Keywords:** multi-disciplinary care, neurocutaneous syndromes, neurofibromatosis, ophthalmic screening, phakomatoses, screening, tuberous sclerosis

## Abstract

Phakomatoses are a heterogenous group of congenital neurocutaneous syndromes that are associated with a wide range of ocular and systemic presentations. The ocular conditions are important to consider as they may be asymptomatic early on, but progress to irreversible vision loss. Due to the variability of disease presentation, timely diagnosis is crucial to preserve visual outcomes. This article provides a comprehensive review of literature on nine major phakomatoses, including Neurofibromatosis type 1, Neurofibromatosis type 2, Tuberous Sclerosis complex, Sturge Weber syndrome, Von Hippel-Lindau, Ataxia Telangiectasia, Basal Cell Nevus Syndrome, Wyburn-Mason syndrome, and PHACE syndrome, which are commonly treated by ophthalmologists and other eye care professionals. Across these syndromes, some common vision threatening manifestation include glaucoma, optic pathway gliomas, and retinal hamartomas, with variability in the age of onset and disease progression. This article aims to provide syndrome specific screening guidelines to allow early detection of ocular pathology and promote multi-disciplinary care among ophthalmologists, pediatricians, and other providers to decrease visual morbidities and improve overall patient outcomes.

## 1 Introduction

Phakomatoses are a broad group of congenital neurocutaneous syndromes with hamartomatous involvement of multiple organ systems, involving the skin, central nervous system, and eyes (Kerrison and Newman, 1999). To date, there are over 60 phakomatoses reported in the literature (Douglas et al., 2019) many of which are associated with distinctive ocular manifestations (Table 1). Among these, patients with nine major syndromes that most commonly present to eye care providers are responsible for most of the ocular symptoms: Neurofibromatosis type 1 (NF1), Neurofibromatosis type 2 (NF2), Tuberous Sclerosis complex, Sturge Weber syndrome, Von Hippel-Lindau, Ataxia Telangiectasia, Basal Cell Nevus Syndrome, Wyburn-Mason syndrome, and PHACE syndrome.

**TABLE 1:** Common phakomatoses associated with ocular manifestations. A summary of major neurocutaneous syndromes that present with ophthalmic involvement, including retinal, optic nerve, and anterior segment findings.

Phakomatosis	Key ocular findings
Neurofibromatosis type 1 (NF1)	Lisch nodules, choroidal nodules, optic pathway glioma, eyelid plexiform neurofibromas, glaucoma
Neurofibromatosis type 2 (NF2)	Posterior subcapsular cataracts, epiretinal membranes, retinal hamartomas, optic nerve sheath meningiomas
Tuberous sclerosis complex (TSC)	Retinal astrocytic hamartomas, achromic retinal patches, retinal pigmentary changes
Sturge–Weber syndrome	Diffuse choroidal hemangioma, glaucoma, episcleral vascular anomalies
Von Hippel–Lindau (VHL)	Retinal capillary hemangioblastomas, exudation, tractional or exudative retinal detachment
Ataxia–telangiectasia	Conjunctival telangiectasia, oculomotor apraxia
Basal cell nevus syndrome	Eyelid basal cell carcinoma, medullated nerve fibers, strabismus, other ocular adnexal tumors
Wyburn-Mason syndrome	Retinal arteriovenous malformations, retinal ischemia, macular edema, neovascular glaucoma
PHACE syndrome	Periocular infantile hemangiomas, optic nerve hypoplasia, coloboma, strabismus
Incontinentia pigmenti	Peripheral retinal vasculopathy, neovascularization, tractional/exudative retinal detachment, optic atrophy, strabismus/nystagmus
Hypomelanosis of Ito	Iris and fundus pigmentary abnormalities, coloboma, strabismus, nystagmus
Klippel-Trenaunay syndrome	Episcleral vascular malformations, choroidal hemangioma, glaucoma
Encephalocraniocutaneous lipomatosis	Epibulbar choristomas, microphthalmia, sclerocornea, anterior chamber anomalies, lid colobomas
Linear nevus sebaceous syndrome	Epibulbar choristomas, limbal dermoids, lid coloboma, retinal and optic disc coloboma, corneal opacities

Although ocular findings may be asymptomatic initially, they can progress to permanent vision loss. For example, glaucoma in Sturge Weber syndrome or ptosis resulting from large plexiform neurofibromas in NF1 are vision threatening. Because these disorders are congenital, many vision threatening manifestations begin in early childhood, emphasizing the importance of age-appropriate and timely ophthalmic screening. The variability of disease presentation combined with the absence of standardized ophthalmic surveillance protocols leads to delayed diagnosis. Given the risk of permanent vision loss and the lack of uniform, evidence-based ophthalmic screening guidelines across syndromes, there is a need for standardized screening recommendations.

The purpose of this guideline article is to summarize the classic systemic and ocular findings of the nine major phakomatoses and to provide screening and management recommendations for eye care providers. In addition, this article recommends interdisciplinary care between primary care providers, ophthalmologists, and other relevant specialists since many of the phakomatoses have multi-organ effects. Early identification of tumors or vascular lesions can prevent development of amblyopia and vision loss and improve long term patient outcomes.

## 2 Neurofibromatosis type 1

### 2.1 Introduction and incidence

Neurofibromatosis type 1 (NF1) is an autosomal dominant disorder that results from pathogenic variants or deletions of the neurofibromin gene located on chromosome 17q11.2 (Jett and Friedman, 2010). It is one of the most common genetic conditions worldwide, with a global prevalence of one in 3,000 individuals and an incidence of one in 2,500 births (Riccardi, 1991; Kallionpää et al., 2018). Although NF1 has a penetrance of close to 100%, its expressivity is highly variable, leading to vastly different phenotypes even within the same family (Jett and Friedman, 2010). Approximately half of cases are *de novo* germline mutations (Jett and Friedman, 2010).

Neurofibromin, the gene product of NF1, is a tumor suppressor gene that is expressed in multiple cells of the body including neurons, glial cells, Schwann cells, and melanocytes. Loss of neurofibromin results in formation of neurofibromas in Schwann cells, café au lait macules in melanocytes, and Lisch nodules in iris, among numerous other findings. Due to its broad phenotypic spectrum, the most updated diagnostic criteria require an individual without an affected parent to have two or more of the following (Legius et al., 2021):.

**FIGURE 1:**
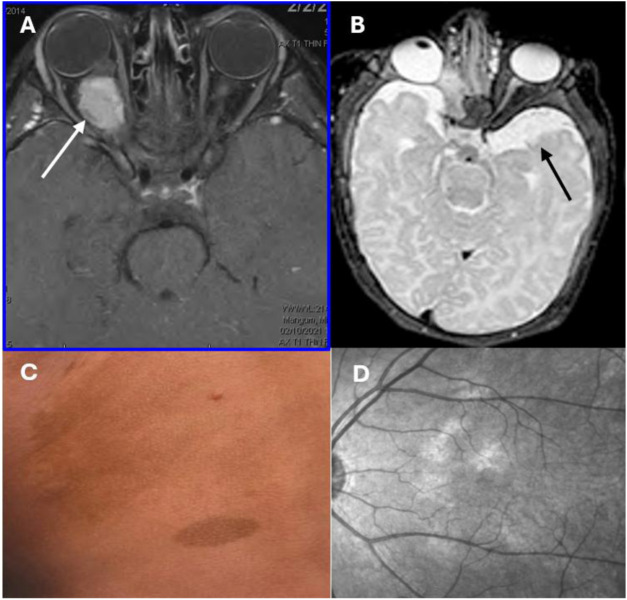
Representative ocular and systemic findings in neurofibromatosis type 1. **(A)** Optic pathway glioma on MRI. **(B)** Sphenoid bone dysplasia. **(C)** Cutaneous neurofibroma and Café-Au Lait spots. **(D)** Choroidal nodules visualized on Near InfraRed OCT.

- axillary/inguinal freckling
- optic pathway glioma (Figure 1A)
- distinctive osseous lesion (Figure 1B)
- six or more café-au-lait macules (>5 mm diameter in prepubertal patients OR >15 mm diameter in post-pubertal) (Figure 1C)
- either two or more Lisch nodules or two or more choroidal abnormalities (Figure 1D)
- presence of a heterozygous pathogenic NF1 variant
- 2 or more neurofibromas or one plexiform neurofibroma.

### 2.2 Systemic features

The systemic features of NF1 commonly include multiple cafe-au-lait macules (>99%), intertriginous freckling (85%), multiple cutaneous neurofibromas, plexiform neurofibromas (30%), malignant peripheral nerve sheath tumor (8%–13%), skeletal dysplasia (long bone dysplasia, dystrophic scoliosis, osteoporosis), hypertension (with higher prevalence in adults compared to children), and learning, behavioral, and/or social difficulties (Ferner et al., 2007; DeBella et al., 2000).

### 2.3 Ophthalmic features

NF1 presents with a wide range of ophthalmic features, which can be categorized into major and minor types. From anterior to posterior, these include eyelid and orbital abnormalities, iris hamartomas, glaucoma related changes, optic pathway gliomas, and choroidal abnormalities.

#### 2.3.1 Eyelid and orbital involvement

Upper eyelid plexiform neurofibromas are an important manifestation of NF1 and can lead to facial disfigurement, ptosis, visual obstruction, and even proptosis and compressive optic neuropathy in severe cases.

#### 2.3.2 Congenital ectropion uvea

Congenital ectropion uvea, an anterior segment anomaly seen in some patients with NF1, is characterized by posterior iris pigment epithelium on the anterior segment of the iris. It is also associated with dysgenesis of the anterior chamber angle and secondary glaucoma. Retrospective case series have shown that NF1 patients with congenital ectropion showed endothelialization of the anterior chamber angle and severe glaucoma (Edward et al., 2012), demonstrating the importance of early detection.

#### 2.3.3 Glaucoma

Glaucoma is seen in approximately one of 300 patients with NF1, and it can be present at birth as a congenital angle abnormality or arise later in life with involvement of the anterior angle (Abdolrahimzadeh et al., 2015). It is commonly unilateral and occurs on the same side as congenital ectropion or ipsilaterally to eyelid plexiform neurofibromas (Edward et al., 2012; Thavikulwat et al., 2019). Early detection is crucial as untreated glaucoma can lead to irreversible vision loss.

#### 2.3.4 Iris hamartomas

Iris hamartomas, also known as Lisch nodules, are comprised of melanocytes, fibroblasts, and mast cells. Although they do not cause visual changes, they are a hallmark diagnostic feature of NF1 seen in over 95% of affected patients.

#### 2.3.5 Optic pathway gliomas

Optic pathway gliomas occur in 10%–15% of NF1 patients and involve the optic nerves, optic chiasm, or optic tracts (Listernick et al., 1997).

#### 2.3.6 Choroidal abnormalities

Choroidal nodules are frequently seen in the posterior segment of NF1 patients (Figure 1D). One cross-sectional study showed that 82% of NF1 affected patients had choroidal abnormalities and another showed these changes in 71% of pediatric NF1 patients (Viola et al., 2012). These choroidal abnormalities are typically asymptomatic and may be detected with OCT or scanning laser ophthalmoscopy using infrared or near-infrared light (Vagge et al., 2015; Moramarco et al., 2018).

#### 2.3.7 Minor ophthalmic findings

Other less common ocular manifestations include a pulsating proptosis, microphthalmos, exophthalmos, conjunctival neurofibroma, hypertrophic corneal nerves, and microvascular retinal abnormalities (Abdolrahimzadeh et al., 2015). Although these are findings are non-specifics, they are important to recognize as undiagnosed NF1 children may first present to their eye care providers.

### 2.4 Segmental neurofibromatosis

Segmental neurofibromatosis is a mosaic type of NF1 caused by post-zygotic pathogenic variants in the NF1 gene. This leads to clinical manifestations that are localized to a specific part of the body (Legius et al., 2021). Because only a subset of cells harbors the mutation, disease expression may be frequently unilateral. Ophthalmic screening is important in patients with segmental neurofibromatosis because they may not fulfill the classic diagnostic criteria for generalized NF1 due to asymmetric or isolated findings.

### 2.5 Screening strategy for ophthalmology

Optic pathway gliomas (OPGs) occur in 15%–20% of children with NF1, most of whom receive a diagnosis before age 7 with a mean diagnosis at 4.5 years of age (Listernick et al., 1997). Because the risk of vision loss is highest in younger children, typically within the first 2 years of life (Fisher et al., 2012), a baseline comprehensive ophthalmic exam by 1 year of age is recommended. For ongoing surveillance, children under age two should receive the most frequent eye examinations due to highest risk of rapid vision loss. Children between the ages of one and eight should receive annual comprehensive eye examinations (Ferner et al., 2007). Older children and adolescents should receive annual eye examinations if their vision is otherwise stable.

Brain MRI may be considered in a subset of patients in the presence of concerning clinical features such as progressive visual loss, new onset proptosis or other neurological signs (Ferner et al., 2007). Table 2 describes the recommended screening strategy for NF1.

**TABLE 2:** Screening guidelines and frequency for NF1. (Reference 13).

Screening	Frequency
Suspected or known NF1 without optic pathway glioma	Annually until 8 years of age Every other year from ages 8 to 18
NF1 with optic pathway glioma confirmed by MRI - ophthalmology screening	Every 3 months during first year after diagnosis Every 6 months from ages 2–8 years Annually from ages 8–18 years
NF1 withoptic pathway glioma confirmed by MRI - neuroimaging	Every 3 months for first year after diagnosis Every 6 months for the next 2 years Annually from ages 3–5 years Subequently based on clinical judgment

#### 2.5.1 Management

Most patients with asymptomatic and clinically stable OPGs can be observed with careful ophthalmic monitoring. Treatment is indicated when patients present with progressive tumors that can cause vision loss, and it includes the use of chemotherapy agents like vincristine or carboplatin. In more rare cases with vision threatening complications, including eyelid plexiform neurofibromas that lead to ptosis, surgery may be considered. Early phase clinical trials using oral MEK inhibitors have shown benefit in patients with progressive low-grade gliomas and plexiform neurofibromas which are present in up to 50% of NF-1 affected individuals (Klesse et al., 2020). These patients should be monitored routinely for retinal changes. A retrospective study by Hummel et al., 2024 notes that 15% (4/45) of patients developed signs of dry eye syndrome not seen at baseline, which included punctate epithelial erosions, blepharitis, and meibomian gland dysfunction (Hummel et al., 2024). Thus, routine anterior exam is warranted to assess for any MEK-inhibitor related changes in addition to routine OCT macula. Multi-disciplinary care with ophthalmology, neurology, genetics, and other relevant specialties is recommended to treat patients effectively.

### 2.6 Neurofibromatosis type 2

#### 2.6.1 Introduction and incidence

Neurofibromatosis Type 2 (NF2) is a phakomatosis characterized by an autosomal dominant mutation of the NF2 gene on chromosome 22q12, which encodes merlin (Rouleau et al., 1993). NF2 is less common than NF1, with an estimated incidence of one in 33,000 to one in 40,000 in the United Kingdom (Evans et al., 1992). The point prevalence in a later UK study was found to be one in 60,000 (Evans DGR. et al., 2010). Despite its low incidence, NF2 demonstrates nearly 100% penetrance by age 60 (Evans et al., 1992). It is crucial to recognize the presenting characteristics of NF2 because hallmark tumors seen in this condition, such as vestibular schwannomas or other CNS tumors, can contribute to hearing loss and balance impairment.

#### 2.6.2 Systemic features

NF2 is characterized by both central and peripheral nervous system tumors. The hallmark findings seen in NF2 include bilateral vestibular schwannomas, meningiomas, ependymomas, and astrocytomas (Asthagiri et al., 2009). Patients may additionally present with spinal schwannomas, which can contribute to both sensory and motor deficits depending on tumor location.

#### 2.6.3 Ophthalmic features

**FIGURE 2:**
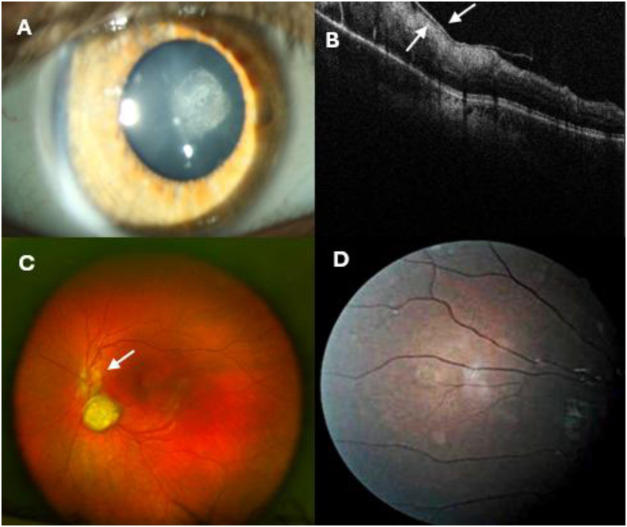
Ocular manifestations in neurofibromatosis type 2 and tuberous sclerosis complex (TSC). **(A)** Posterior subcapsular cataract in NF2. **(B)** Epiretinal membrane in NF2 seen on OCT. **(C)** Color fundus photograph showing calcified retinal astrocytic hamartoma in TSC. **(D)** Hypopigmented retinal lesions in TSC.

On ophthalmic exam, NF2 patients present with a wide range of findings. One cross sectional study (n = 49) reported that cataracts were the most common ocular abnormality in NF2 patients and reported a total frequency of 67% (Ragge and Baser, 1995). Another study confirmed the presence of juvenile posterior subcapsular cataracts in NF2 patients (McLaughlin et al., 2007). Figure 2A shows an example of posterior subcapsular cataract in a patient with NF2, which has a typical age of onset under 30 years of age.

Other ophthalmic manifestations seen in NF2 include retinal hamartomas and epiretinal membranes (ERM). An example of ERM in a patient with NF2 is shown in Figure 2B. In one prospective study, seven out of 9 NF2 patients had posterior pole epiretinal membranes, and five out of nine patients had either posterior cortical or posterior subcapsular cataracts (Kaye et al., 1992). A larger cohort similarly reported the presence of ERMs and retinal hamartomas being frequent ophthalmic findings.

Optic nerve sheath meningiomas (ONSM) are another important ocular manifestation seen in NF2 patients. One observational study demonstrated ONSM in eight out of 30 NF2 patients, which included unilateral (n = 6) and bilateral (n = 2) involvement (Bosch et al., 2006). Though more rarely seen, the presence of ONSM should caution physicians to consider NF2 in their differential and initiate a comprehensive evaluation.

#### 2.6.4 Ophthalmic surveillance after NF2 diagnosis

Ophthalmic screening for NF2 patients should include a baseline comprehensive ophthalmic exam with slit-lamp evaluation for PSC cataracts and a dilated fundus examination to assess for ERMs and retinal hamartomas. After baseline exam, annual follow up with slit lamp and fundus examinations are recommended. When ERM is suspected or visual acuity changes occur, ophthalmologists should also obtain OCT.

#### 2.6.5 Management

Management should be targeted to the specific ophthalmic findings seen in individual patients. In patients with cataracts, extraction is advised if the patient is symptomatic. For instance, juvenile PSC may be symptomatic and require removal. For ERMs, management is based on severity. For mild ERM, observation with OCT is recommended. For moderate to severe cases, vitrectomy with membrane peel may be indicated if ERM causes tractional distortion of the macula or vision loss. Given the high likelihood of concurrent hearing loss due to vestibular schwannomas, many NF2 patients may benefit from referrals to vision rehabilitation services and communication support services.

### 2.7 Tuberous sclerosis

#### 2.7.1 Introduction and incidence

Tuberous sclerosis complex (TSC) is an autosomal dominant multisystemic disorder caused by pathogenic variants in TSC1 (9q34; hamartin) or TSC2 gene (16p13.3; tuberin). The hamartin-tuberin complex negatively regulates mTOR signaling and loss of function leads to widespread hamartoma formation (Northrup and Krueger, 2024). The incidence of TSC ranges from one in 6,000 to one in 10,000 individuals and approximately two-thirds of cases arise from *de novo* mutations (Northrup and Krueger, 2024).

#### 2.7.2 Systemic features

TSC affects multiple organ systems, most commonly the central nervous system, heart, kidneys, and skin. Systemic manifestations include seizures, cortical or subependymal tubers, subependymal giant cell astrocytoma, cardiac rhabdomyomas, renal angiomyolipomas, and facial angiofibromas.

#### 2.7.3 Ophthalmic features

Ophthalmic findings in TSC can be categorized into major and minor types. Major features include retinal astrocytic hamartomas (Figure 2C), with prevalence estimates ranging from 40%–50% (Rowley et al., 2001). These lesions arise from the posterior pole or along the retinal nerve fiber layer. Retinal hamartomas are morphologically classified into three main types: 1) flat, smooth, non-calcified lesions that are translucent; 2) elevated, multinodular, calcified lesions that are opaque, and 3) mixed lesions with features of both. Population based studies have shown that the flat, smooth, non-calcified type of hamartoma is most common and there was no correlation between age and presence of retinal astrocytic hamartomas overall (Rowley et al., 2001).

These lesions typically have a minimal effect on visual acuity; however, visual impairment may occur when the hamartomas involve the macula or have associated exudation or subretinal fluid which can result in secondary macular edema or exudative retinal detachment (Shields et al., 2004). Rare instances of hamartomas causing vitreous hemorrhage have been reported, but these lesions are typically larger and more progressive (Atkinson et al., 1973). These findings underscore the need to assess the location, size, and associated features for determining visual prognosis.

Minor features include retinal pigmentary changes which appear hypopigmented (Figure 2D) or “punched out” in appearance especially in the posterior pole or midperiphery. Other findings include palpebral angiofibromas of the eyelid, iris or choroidal colobomas, and iris depigmentation (Hodgson, 2017). While these findings may be asymptomatic, they are important markers of TSC and contribute to visual changes.

#### 2.7.4 Screening strategy for ophthalmology

Since retinal astrocytic hamartomas are commonly seen in TSC patients and may sometimes progress, we recommend a baseline comprehensive ophthalmic examination at the time of diagnosis, followed by annual surveillance. Routine monitoring should include a dilated fundus examination, OCT to assess for subretinal fluid or macular involvement, and fluorescein angiography when lesions show exudation, other atypical features, or progressive changes (Hodgson, 2017).

Retinal pigmentary changes should be monitored with routine fundus examination. To assess for presence of palpebral angiofibroma, iris depigmentation and iris or choroidal coloboma, periodic monitoring with slit lamp examination is recommended.

#### 2.7.5 Management

Most retinal astrocytic hamartomas are stable and require observation only. However, if patients present with exudative lesions or choroidal neovascularization, treatment may be indicated. A couple case reports have suggested that intravitreal anti-VEGF therapy, such as bevacizumab, may reduce macular edema exudation associated with tuberous sclerosis (Nakayama et al., 2012; Saito et al., 2010). However, the role of anti-VEGF in treating retinal astrocytic hamartomas is limited to smaller studies, so larger studies are needed to determine its efficacy and long-term safety. Alternatively, systemic mTOR inhibition with drugs such as everolimus may be considered in patients with progressive or vision threatening astrocytic hamartomas, especially when there are concurrent systemic indications for therapy such as renal angiomyolipomas (Krueger et al., 2010; Bissler et al., 2013; Franz et al., 2015). Multidisciplinary coordination is highly recommended when administering systemic therapy to patients.

### 2.8 Sturge-Weber syndrome

#### 2.8.1 Introduction and incidence

Sturge-Weber Syndrome is a neurocutaneous disorder characterized by a somatic gain-of-function mutation in the GNAQ gene located on chromosome 9q21.2., which disrupts normal vascular development in the orbital, cerebral and facial regions (Shirley et al., 2013). Disease presentation is characterized by port-wine birthmarks, neuronal changes, and malformation of capillary vasculature. This condition is estimated to affect approximately one in every 20,000 to 50,000 newborns globally with equal presentation in males and females (Higueros et al., 2017).

#### 2.8.2 Systemic features

The clinical presentation of Sturge-Weber syndrome is multisystemic. Neurologic manifestations appear in 70%–80% of patients with many symptoms emerging during infancy. One of these manifestations includes capillary-venous leptomeningeal malformation, which consists of tortuous, dilated capillary vessels overlying the occipital or parietal lobes. The underlying cerebral tissue may become ischemic and exhibit atrophy or calcification, as seen in studies identifying increased fibronectin, laminin, and type IV collagen staining in affected regions (Comi, 2003). These changes present in infants as epilepsy with seizures and stroke-like episodes. Additional neurological features may include migraines and developmental delays.

**FIGURE 3:**
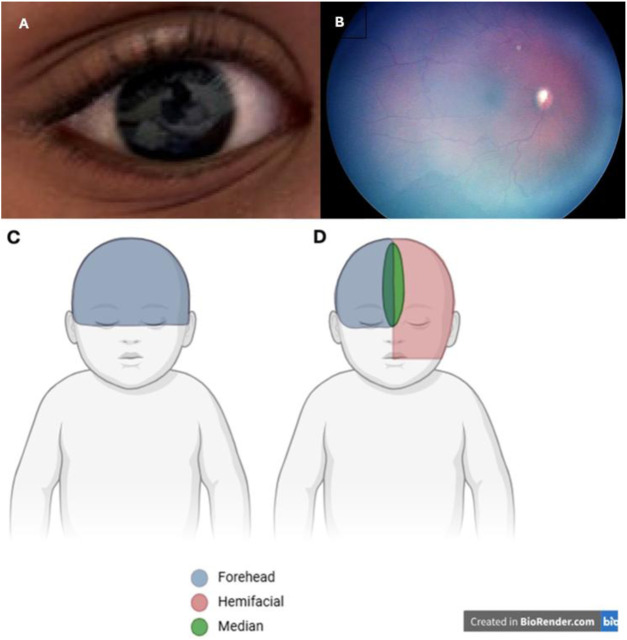
Ocular Manifestations in Sturge Weber Syndrome. Conjunctival erythema and buphthalmos from Sturge Weber **(A)** and fundus appearance of diffuse choroidal hemangioma **(B)** and dermatome distribution of port wine stain **(C,D)** (modified from Sabeti et al., 2021).

Cutaneous presentations of Sturge-Weber include conjunctival erythema and buphthalmos (Figure 3A) in addition to vascular malformations of the facial region, typically seen as a port-wine birthmark, often following trigeminal nerve distribution. Previous studies have categorized port-wine stains by varying facial region, as seen in Figures 3C,D. Other systemic manifestations of Sturge-Weber have been noted, including endocrine abnormalities such as hypothyroidism and growth hormone deficiency (Comi, 2015).

#### 2.8.3 Ophthalmic features

Glaucoma is the most common ocular manifestation of Sturge-Weber Syndrome, affecting 30%–70% of patients (Hassanpour et al., 2021). Evidence suggests that the distribution of port-wine stains may influence risk of neonatal glaucoma, with one study showing involvement of the S2 region or lower eyelids in newborns linked to an increased risk of developing glaucoma (Ha et al., 2020).

Choroidal hemangioma is also prevalent in 40%–50% patients with Sturge-Weber Syndrome and is described as having a “tomato ketchup” appearance on fundoscopy (Figure 3B). Patients may present with refractive changes, retinal detachments, or changes in the retinal pigment epithelium involving the macula (Hassanpour et al., 2021).

Manifestations of Sturge-Weber syndrome overlap or may present concurrently with Phakomatosis Pigmentovascularis (PPV), sharing characteristics of vascular malformation such as port wine stains and increased risk of glaucoma. PPV has other unique characteristics including nevi and Mongolian spots, hyperpigmentation of the sclera, iris, or conjunctiva, and increased risk of uveal melanoma (Abdolrahimzadeh et al., 2015).

#### 2.8.4 Screening strategy in ophthalmology

Port-wine birthmarks are present at birth in infants and necessitate ophthalmic screenings every few months from infancy into early childhood, including glaucoma screening and indirect retinoscopy. Additional imaging, including A-scan and B-scan ultrasounds, may be warranted when fundus abnormalities are observed to aid in the diagnosis of choroidal hemangioma (Hassanpour et al., 2021). Recent studies have outlined how the use of optical coherence tomography (OCT) may also be used to enhance this diagnosis. One study demonstrated that swept-source OCT (SS-OCT) revealed characteristic reductions in choroidal thickness and alterations in choroidal vascular architecture (Surve et al., 2019). Other studies have utilized enhanced depth imaging OCT (EDI-OCT) for diagnosis of choroidal hemangioma, noting a smooth, sloping mass with expansion of choroidal vessels (Shields et al., 2015). Ongoing ophthalmologic surveillance is recommended throughout adolescence and adulthood, even when early examinations reveal no signs of ocular involvement (Comi, 2015).

#### 2.8.5 Management

Management of Sturge-Weber Syndrome requires a multidisciplinary approach to address the dermatologic, ophthalmic, neurologic, and endocrine symptoms that a patient may present with. Early detection of glaucoma in patients is essential in preserving visual function, with some studies suggesting that a combination of medical and surgical based treatment may be most effective. Topical agents, including latanoprost, topical propranolol and carbonic anhydrase inhibitors have shown to be effective in reducing intraocular pressure. Surgical interventions, including trabeculectomy, trabeculotomy–trabeculectomy combination procedure, and other drainage procedures, should be considered when medical interventions are insufficient (Silverstein and Salvin, 2019). Photodynamic therapy continues to be supported as a treatment for choroidal hemangiomas.

Port-Wine birthmarks can be treated in infancy with laser therapy to improve cosmetic outcomes and limit progression, with some studies suggesting that laser treatment earlier in life (below 6 months of age) may decrease the number of treatment sessions required. Patients experiencing neurologic changes, particularly seizures, should be managed with anti-convulsant or surgical intervention for refractory cases (Chapas et al., 2007). Due to an increased prevalence of endocrine abnormalities in Sturge-Weber patients, including growth hormone deficiency and central hypothyroidism (Bachur and Comi, 2013), regular endocrine screening should be performed with symptoms treated as indicated.

### 2.9 Von Hippel-Lindau

#### 2.9.1 Introduction and incidence

Von Hippel-Lindau (VHL) disease is an inherited multifactorial disease affecting both the central nervous system and various organs. It results from an autosomal dominant loss of function mutation in the VHL gene located on chromosome 3p25-26, a tumor suppressor gene that plays a key role in cellular adaptation in hypoxic environments (Rana et al., 2021). VHL has a prevalence ranging from one in 35,000 to one in 91,000 individuals (Evans DG. et al., 2010) with clinical manifestations typically appearing at the second decade of life (Chittiboina and Lonser, 2015).

Diagnostic criteria of VHL are based on characteristic clinical findings and depend on whether the patient has a positive family history. Diagnostic criteria are summarized in Table 3.

**TABLE 3:** Diagnostic Criteria for Von Hippel Lindau Disease (Reproduced from Khan, 2021) stratified by a positive or negative family history for VH.

Family history	Diagnostic criteria
Positive	Diagnosis requires one or more of the following: Two or more of the hemangioblastomas of the retina or central nervous system One hemangioblastoma of the retina or central nervous system and one other associated manifestation (listed 1–4) 1. Renal cell carcinoma 2. Endolymphatic sac tumor 3. Pheochromocytoma, paraganglioma, or glomus tumor 4. Pancreatic neuroendocrine tumor or pancreatic cysts
Negative	Diagnosis requires one or more of the following: One pathogenic variant in VHL (in the patient or a first degree relative) and a hemangioblastoma of the retina or central nervous system One pathogenic variant in VHL (in the patient or a first degree relative) and one of the diagnostic criteria (listed 1–4 above)

#### 2.9.2 Systemic features

Central nervous system hemangioblastomas are often the first manifestation of VHL, commonly affecting the cerebellum and spinal cord. Early symptoms may include neurologic changes such as weakness, paresthesia, ataxia, and slurred speech (Gläsker et al., 2020). Endolymphatic sac tumors are also commonly observed in VHL patients, with bilateral hearing loss representing the earliest symptom (Manski et al., 1997).

Patients diagnosed with renal cell carcinoma are reported to experience variable symptoms due to varying tumor growth rates, including lower back pain and hematuria (Bausch et al., 2013). In contrast, patients with pancreatic manifestations and pheochromocytomas are most commonly asymptomatic (Gläsker et al., 2020).

#### 2.9.3 Ophthalmic features

Retinal capillary hemangiomas are among the most common presentations in VHL, affecting around 60% of affected individuals (Khan et al., 2021) with bilateral involvement in half of patients (Magee et al., 2006). The risk of development increases with age and tends to plateau at age 30 (Webster et al., 1998) with the mean age at diagnosis of about 25 years (Chang et al., 1998). While many patients are asymptomatic during early disease, visual impairment can occur with further progression. One study reported 8% of VHL patients with vision at 20/200 or worse (Chew, 2005). Another study identified that VHL may cause blindness in 15% of affected eyes (Wittström et al., 2014).

**FIGURE 4:**
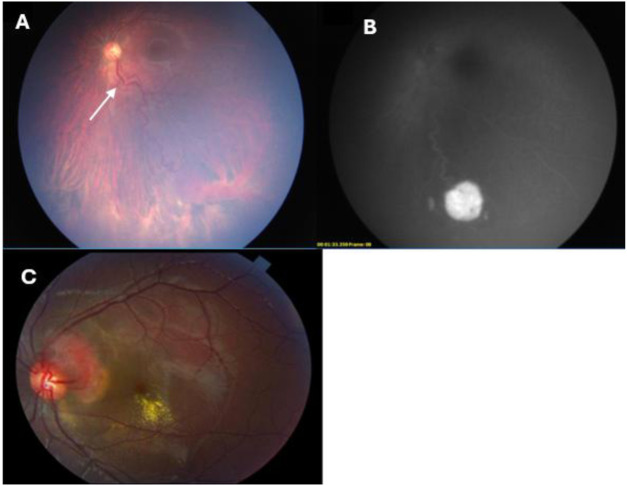
Ocular Manifestations in Von Hippel Lindau. Peripheral retinal hemangioblastoma with dilated feeder vessel **(A)** showing leakage on fluorescein angiography **(B)**. Juxta-papillary hemangioblastoma with macular exudation **(C)**.

On exam, retinal capillary hemangiomas are commonly seen in the periphery as a round, orange-red tumor with prominent vasculature (Figure 4). They may also be found within the juxtapupillary region, temporal of the optic disc, in around 11%–15% of VHL cases (Singh et al., 2001). Tumors located in this region can present as three different forms. In the exophytic form, tumors appear as an orange-colored lesion, similar to chronic optic disc edema. The endophytic form protrudes from the optic disc into the vitreous cavity. In contrast to these, the sessile form is difficult to diagnose due to its subtle appearance. All three forms may contribute to further retinal damage including retinal detachments and macular damage from tumor fluid exudation (Khan et al., 2021).

#### 2.9.4 Screening strategy in ophthalmology

Early surveillance is critical for timely detection and management of symptoms, especially in patients with a known family history of VHL. According to one consensus guideline, children who have a known family history of VHL or suspected VHL disease should have frequent dilated ophthalmic screenings early in life, with genetic testing recommended for high risk individuals. For individuals diagnosed with VHL, exams are recommended to start at 12 months of age and continue yearly to twice a year until the age of 30, when appointments can be moved to annual. Frequency of visits can be modified based on quality and findings of the exam. Dilated funduscopic exams are a critical part of early examination, but ultra-widefield photography and ultra-widefield angiography may be valuable tools for diagnosis of retinal hemangiomas (Daniels et al., 2023). OCT is another useful resource for evaluating retinal thickness, delineating lesion dimensions, and for identifying additional complications such as a retinal detachment (Khan et al., 2021).

#### 2.9.5 Management

Ophthalmologic management varies depending on severity of lesions and treatment response. Laser photocoagulation and cryotherapy are the recommended first line treatments in small tumors, while larger tumors may require a vitrectomy (Singh et al., 2002). Belzutifan, an oral small molecule HIF2-α inhibitor, is an FDA approved drug in treating tumors associated with VHL, and some studies show promising results in treating ocular changes in VHL disease (Jamshidi et al., 2025). Additional treatment modalities, such as anti-VEGF agents and radiation therapy are another useful resource used to reduce tumor size and prevent progression (Glasker et al., 2001). Given the multisystem presentation of VHL, it is recommended to use a multidisciplinary treatment to address potential CNS tumors or organ lesions (Louise et al., 2022).

### 2.10 Ataxia telangiectasia

#### 2.10.1 Introduction and incidence

Ataxia telangiectasia (A-T) is an autosomal recessive disorder characterized by primary immunodeficiency with an increased risk of malignancy, respiratory, and endocrine complications (Collyer and Rajan, 2024). It is caused by a mutation in the ATM gene, located on chromosome 11q22-23 (Gatti et al., 1988), which encodes for a protein that is responsible for signaling pathways that respond to stress caused by DNA double strand breaks. A-T has a global prevalence estimated to be one in 40,000 to one in 100,000 live births (Rothblum-Oviatt et al., 2016).

#### 2.10.2 Systemic features

Due to its multisystemic manifestations, including cerebellar degeneration, respiratory manifestations, premature aging and endocrine dysfunction, the life expectancy of individuals with classical A-T is often limited to 20–30 years (Petley et al., 2022). Neurologic symptoms have a gradual onset, starting around 6–18 months of age and becoming more pronounced during early school years. Common manifestations of neurological changes include gait ataxia and dysphagia, with most individuals requiring a wheelchair around 10 years old (Aguado et al., 2022). Endocrine dysfunction in A-T presents as poor growth, delayed puberty (Rothblum-Oviatt et al., 2016), and insulin resistance (Europe, 2016). Immune dysfunction is another hallmark trait of patients with A-T, as seen with impaired humoral immune system and decreased production of T lymphocytes, which substantially increases the risk of developing malignancies (Nowak-Wegrzyn et al., 2004).

#### 2.10.3 Ophthalmic features

Commonly occurring ophthalmic features include oculocutaneous telangiectasia, which may appear as conjunctivitis, and involvement of the bulbar conjunctiva (Rothblum-Oviatt et al., 2016). These findings do not directly affect visual acuity, but other manifestations can still be seen in the form of ocular motor defects. These may include impairment of convergence and gaze holding and prolonged saccade latency, which is noted to worsen with increasing age (Lewis et al., 1999). Other ocular findings include nystagmus and variable strabismus (Tang and Shaikh, 2018).

#### 2.10.4 Screening strategy in ophthalmology

Many studies highlight that ocular changes may play a vital role in overall diagnosis. In one study, 91% of A-T patients had anterior oculocutaneous telangiectasia and bulbar conjunctival involvement, while no posterior vasculature abnormalities were reported (Farr et al., 2002). These findings underscore the importance of periodic anterior eye exams beginning at a young age, particularly in individuals who also present with systemic symptoms of A-T.

When these ocular changes are observed, further evaluation is recommended. Patients with multiple A-T symptoms or a known family history should undergo genetic testing and counseling (Rothblum-Oviatt et al., 2016).

#### 2.10.5 Management

As A-T has no known treatment or cure, disease management focuses on supportive care. Specific ocular intervention may include reading aids or correction of strabismus through surgical procedures or lenses with a prism add (Farr et al., 2002). Additionally, some individuals may experience photophobia and benefit from filtered lenses (Riise et al., 2007).

Other management such as physical therapy, antibiotics to combat infections, feeding accommodations, and cancer screenings should also be considered for systemic monitoring and management as needed (Rothblum-Oviatt et al., 2016).

### 2.11 Wyburn Mason

#### 2.11.1 Introduction and incidence

Wyburn Mason Syndrome is a rare congenital disorder characterized by spontaneous vascular malformations primarily affecting the brain, ocular, and skin of the facial regions. Patients present with arteriovenous malformations (AVM) of varying sizes, with symptoms typically emerging in the second or third decade of life as the AVMs progress, as smaller AVMs do not produce any notable symptoms (So et al., 2023). The average age of diagnosis is noted to be 15.6 years (Shameem et al., 2024).

#### 2.11.2 Systemic findings

AVMs affecting the brain most often involve midbrain on the same side as the affected eye; however, location of the AVM may determine a patient’s presentation and symptoms. Neurologic manifestations can include headaches, seizures, and hemorrhage. Cutaneous lesions are rare but present in a subset of Wyburn Mason patients, manifesting as angiomas of the facial region (Ponce et al., 2001), often following a trigeminal nerve pattern (So et al., 2023).

#### 2.11.3 Ophthalmic features

Ophthalmic changes are typically the first symptoms of Wyburn Mason syndrome and are characterized by AVMs localized in the retina. These lesions typically affect one eye, but cases of bilateral involvement have been reported (Shameem et al., 2024). Presence of a large AVM may cause retinal degeneration or compression of the optic nerve leading to hemorrhage or ischemia. Patients report this as sudden or gradual decreased vision. Proptosis and glaucoma may also be noted in some patients (Ponce et al., 2001).

**FIGURE 5:**
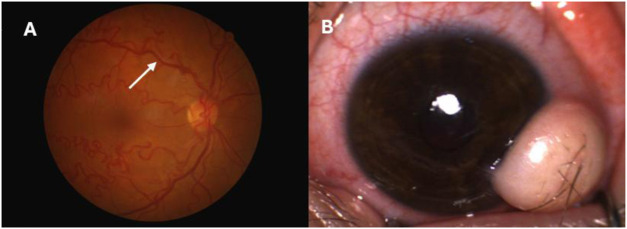
**(A)** Ocular Manifestations in Wyburn Mason with tortuous vasculature. **(B)** Limbal dermoid in Linear Nevus Sebaceous Syndrome.

On exam, the fundus of a Wyburn Mason patient with retinal AVM appears with dilated, tortuous vessels stemming from the optic disc and extending across the retina (So et al., 2023). This can be seen in Figure 5A.

#### 2.11.4 Screening strategy in ophthalmology

Exams involving retinoscopy can help to visualize the fundus and diagnose Wyburn Mason. Additional visualization of vasculature may be done with fluorescein angiography, which demonstrates rapid filling of vascular anomalies without significant leakage and may be a useful resource for identifying small lesions. Angiography shows demonstrates rapid filling of vascular anomalies without significant leakage which is a particularly useful resource for identifying small lesions. Long-term monitoring of macular thickness, progression, or further complications such as hemorrhaging is recommended with OCT and ultrasound imaging (So et al., 2023).

#### 2.11.5 Management

Management of Wyburn Mason depends on the size and location of AVM. If unruptured and not obstructing vision, ophthalmic monitoring should be maintained. If further complications arise, such as glaucoma, retinal photocoagulation or vitrectomy may need to be considered. Brain imaging should be one to confirm the presence and size of a lesion when ocular lesions are identified (So et al., 2023).

### 2.12 PHACE syndrome

#### 2.12.1 Introduction and incidence

PHACE Syndrome, an acronym for posterior fossa anomalies, hemangioma, arterial lesions, cardiac abnormalities/coarctation of the aorta, eye anomalies, is a sporadic disorder characterized by large facial hemangiomas and congenital anomalies (Frieden et al., 1996). Although the exact cause unknown, studies have outlined that mothers of children with PHACE had a significantly higher rate of pregnancy complications, like preeclampsia, and placental abnormalities such as placenta previa (Wan et al., 2017). Diagnostic criteria are listed below in Table 4.

**TABLE 4:** Diagnostic criteria for PHACE syndrome. Modified from (Garzon et al., 2016).

Diagnostic criteria	Arterial anomalies	Brain structure	Cardiovascular	Ocular	Ventral
Major criteria	Anomaly of major cerebral or cervical arteries Dysplasia of the large cerebral arteries Arterial stenosis or occlusion with or without moyamoya collaterals Absence or moderate-severe hypoplasia of the large cerebral and cervical arteries Aberrant origin or course of the large cerebral or cervical arteries except common arch variants such as bovine arch Persistent carotid-vertebrobasilar anastomosis (proatlantal segmental, hypoglossal, otic, and/or trigeminal arteries)	Aneurysm of any of the cerebral arteries	Aortic arch anomalies Coarctation of the aorta Dysplasia Aneurysm Aberrant origin of the subclavian artery with or without a vascular ring	Posterior segment abnormalities Persistent hyperplastic primary vitreous Persistent fetal vasculature Retinal vascular anomalies Morning glory disc anomaly Optic nerve hypoplasia Peripapillary staphyloma	Anomaly of the midline chest and abdomen - Sternal defect - Sternal pit - Sternal cleft - Supraumbilical raphe
Minor criteria	Aneurysm of any of the cerebral arteries	Midline brain anomalies Malformation of cortical development	Ventricular septal defect Right aortic arch/double aortic arch Systemic venous anomalies	Anterior segment abnormalities Microphthalmia Sclerocornea Coloboma Cataracts	Ectopic thyroid hypopituitarism Midline sternal papule/hamartoma
Possible diagnosis	Hemangioma >5 cm in diameter of the head including scalp PLUS 1 minor criteria Hemangioma of the neck, upper trunk or trunk and proximal upper extremity PLUS 1 major or 2 minor No hemangioma PLUS 2 major criteria				
Definitive diagnosis	Hemangioma >5 cm in diameter of the head including scalp PLUS 1 major criteria or 2 minor criteria Hemangioma of the neck, upper trunk or trunk and proximal upper extremity PLUS 2 major criteria				

#### 2.12.2 Systemic findings

Cutaneous abnormalities are a defining feature of PHACE Syndrome, most notably with the characteristic hemangioma of the facial and cervical region. Limbal demoids are also a hallmark finding in this condition (Figure 5B). One study showed 91% of infants with PHACE Syndrome presenting with infantile hemangioma, underscoring this hallmark feature (Garzon et al., 2016). Neurological manifestations consist of structural abnormalities and include posterior fossa malformations and Dandy-Walker formations (Metry et al., 2009). Vascular manifestations of PHACE Syndrome include congenital cerebral vascular anomalies, which were identified in 77.4% of patients from one study subset (Heyer et al., 2008). Cardiovascular anomalies, including ventricular and septal defects, are found to occur in 21%–67% of PHACE patients (Bayer et al., 2013). Dental abnormalities, dysphagia, developmental delays, and thyroid dysfunction have also been noted (Keith, 2024).

#### 2.12.3 Ophthalmic findings

Ophthalmic manifestations, including microphthalmia, morning glory disc appearance, persistent fetal vasculature, and optic nerve atrophy as the most common, typically present on the same side as the facial hemangiomas (Metry et al., 2009). Additional ocular complications, amblyopia, strabismus, ptosis, have also been reported (Kronenberg et al., 2005). Other rare abnormalities have also been described in the literature including retinal vasculature anomalies, iris vessel hypertrophy, coloboma, and cataracts (Metry et al., 2009).

#### 2.12.4 Ophthalmic screening

In cases of possible PHACE Syndrome, a thorough eye exam is recommended with only continued follow up for patients with abnormal exam findings. In situations of abnormal exam, frequency of follow ups is determined by a child’s risk of developing amblyopia or additional complications (Soliman et al., 2022).

#### 2.12.5 Management

Overall primary management focuses on treating infantile hemangiomas, monitoring cardiovascular function closely, and following neurological status for changes (Keith, 2024). Management of ocular abnormalities depends largely on the extent of involvement. Early ophthalmic monitoring is essential, with medical interventions aimed at treating amblyopia or refractive error. Surgical intervention may also be necessary in cases such as ptosis, strabismus, or microphthalmia, helping to preserve visual acuity. Treatment of cutaneous hemangioma with beta blockers may also help to reduce the spread and involvement of the periocular region (Soliman et al., 2022).

## 3 Discussion

Many of the phakomatoses discussed in this article, including NF1, NF2, TSC, Sturge-Weber Syndrome, VHL, and A-T have genetic etiologies involving dysregulation of signaling pathways. These often result in overlapping phenotypic changes that can be observed on clinical appearance or ophthalmic exam. Several of the phakomatoses demonstrate vascular changes, such as vascular malformation or tortuous vessels, and can be observed in Wyburn Mason Syndrome and VHL. Optic nerve abnormalities are observed in both NF1 and PHACE Syndrome. Other changes in ocular and cutaneous appearances, such as iris hamartomas, port wine stains, hemangiomas, and oculocutaneous telangiectasia are suggestive of conditions such as Sturge Weber Syndrome, A-T, and NF-1.

Given these overlapping changes, a unified screening framework may be developed to ensure early detection and intervention. Children with suspected neurocutaneous syndromes should undergo a comprehensive ophthalmic evaluation by a pediatric ophthalmologist, including a dilated fundus exam, refraction, and ocular imaging such as OCT or fundus photography. If a child presents with characteristic changes of phakomatoses, a multidisciplinary approach should be taken including a referral to genetics and relevant specialties for diagnosis and management.

Recent advances in ophthalmic imaging have significantly improved early detection and monitoring of the ocular manifestations associated with the phakomatoses. OCT has been used to evaluate retinal changes in many neurocutaneous syndromes. For instance, in tuberous sclerosis, OCT allows for high resolution visualization of retinal astrocytic hamartomas and any associated findings such as subretinal fluid. It is also helpful in visualizing the epiretinal membranes seen in NF2 and identifying other macular changes that may not be seen easily on fundus examination. Early detection of these ocular signs may allow for earlier intervention and closer monitoring of complications such as exudative retinal detachment.

OCT angiography is another advancement in imaging that is helpful for dissecting flow in the different layers of the retina. This imaging is especially helpful for the phakomatoses that present with vascular changes, such as von-Hippel Lindau and Wyburn-Mason syndrome.

Finally, ultra-wide field retinal imaging has also expanded the ability to detect retinal pathologies that are located more peripherally. This is especially helpful for conditions like von-Hippel Lindau where peripheral retinal capillary hemangiomas are common and early detection is critical to prevent vision threatening complications.

Finally, outside of imaging modalities, artificial intelligence may play an important role in screening and risk stratification. For example, AI based imaging has recently been used in identifying retinal pathologies such as diabetic retinopathy and macular edema (Teng et al., 2025). These technologies may be helpful for identifying retinal changes associated with neurocutaneous syndromes and should be explored with future studies.

One major limitation is the lack of longitudinal studies that evaluate the course of the ocular findings across the nine discussed phakomatoses. Because of this, most current screening recommendations are based on smaller studies. In addition, inconsistencies remain in terms of recommended screenings. For example, the optimal timing and frequency of neuroimaging in NF1 is debated; while some experts recommend imaging only in patients who are symptomatic, others encourage earlier imaging to allow for timely intervention in these patients.

Another important unmet need is the development of standardized multidisciplinary protocols that integrate care among ophthalmologists, neurologists, genetics, dermatology, and primary care providers. Because the phakomatoses have multi-system involvement, a coordinated model may improve early detection and follow up care in patients.

## 4 Conclusion

While many of the phakamotoses described in this guideline article, including Wyburn-Mason, PHACE syndrome, and A-T have been explored in literature, this is the first comprehensive review that collectively describe the nine major phakomatoses eye care professionals treat. As there is limited data on longitudinal monitoring, future research should be aimed at investigating standardized screening guidelines, conducting prospective longitudinal studies, and assessing the role of emerging technologies on earlier disease detection rates and monitoring. Advances in imaging may increase the ability to detect subtleties on ocular imaging and allow for better risk stratification of patients.

## Data Availability

The original contributions presented in the study are included in the article/supplementary material, further inquiries can be directed to the corresponding author.
